# Exploring the Most Effective Strategy for Purine Metabolite Quantification in Veterinary Medicine Using LC–MS/MS

**DOI:** 10.3390/vetsci12030230

**Published:** 2025-03-03

**Authors:** Anisa Bardhi, Francesco Dondi, Andrea Barbarossa

**Affiliations:** 1 Department of Veterinary Medical Sciences, Alma Mater Studiorum—University of Bologna, Ozzano dell’Emilia, 40064 Bologna, Italy; f.dondi@unibo.it (F.D.); andrea.barbarossa@unibo.it (A.B.); 2 Health Sciences and Technologies—Interdepartmental Centre for Industrial Research (CIRI-SDV), Alma Mater Studiorum—University of Bologna, Ozzano dell’Emilia, 40064 Bologna, Italy

**Keywords:** allantoin, bovine, clinical pathology, dog, nutrition, serum, standard addition, uric acid, urine

## Abstract

Measuring purine metabolites in the serum and urine may help in the early diagnosis of urolith formation, the correction of allopurinol therapeutic dosages, and the evaluation of diet-related alterations. To properly monitor these endogenous compounds and assess their physiological concentration ranges in biological fluids, highly specific and accurate analytical approaches are required. Colorimetric assays are generally used for this purpose, although their cross-reactivity could lead to incorrect determinations. Given the importance of selectivity in detecting endogenous compounds, this study explored the use of liquid chromatography–tandem mass spectrometry (LC–MS/MS) to enhance uric acid and allantoin measurement in both serum and urine from dogs, as well as urine from bovines. The most effective analytical strategy was identified and successfully applied to an initial batch of samples collected from healthy dogs and bovines. In conclusion, mass spectrometry proved to be a powerful tool for this challenging task, further demonstrating its superior performance in improving clinical laboratory diagnostics, including its valuable applications in veterinary medicine.

## Simple Summary

Determining the optimal method for purine metabolite quantification is crucial in veterinary medicine. Uric acid and allantoin, in particular, serve as potential biomarkers in both dogs and bovines. Their association with various pathologies and their sensitivity to dietary influences make them particularly relevant for nutritional interventions in both small and large animals.

## 1. Introduction

**Figure 1:**
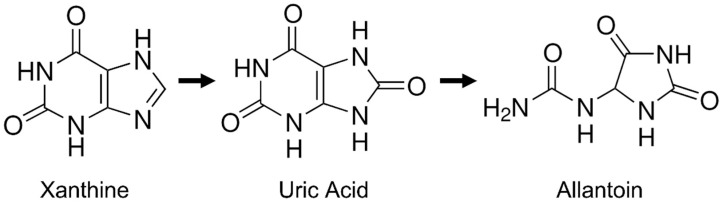
Final steps of purine metabolism: xanthine is oxidized to uric acid, which is subsequently converted to allantoin.

Purines are catabolites derived from the endogenous breakdown of cells, DNA and RNA, whose metabolism also depends on the individual’s genetics, diet and lifestyle [1,2]. The primary site for purine metabolism (see Figure 1) is the liver, and the resulting molecules are mostly excreted in the urine. The hepatic enzyme xanthine oxidase catalyzes the conversion of hypoxanthine to xanthine, which is then further oxidized to uric acid—the primary end product of purine degradation in humans and higher primates [1,2,3,4]. In dogs, the urate oxidase enzyme plays a relevant role in the further conversion of uric acid to allantoin, which is more soluble in urine [5]. However, Dalmatians and other canine breeds (e.g., English Bulldogs) have an autosomal inherited defect in uric acid transport to hepatic cells, which can lead to hyperuricosuria [6,7]. This condition is often treated with low purine diets and allopurinol, a xanthine dehydrogenase inhibitor that reduces urate urolith formation. Moreover, allopurinol is frequently used in dogs for the long-term treatment of leishmaniasis [8]. If improperly administered, allopurinol can increase the xanthine concentration in urine, leading to the development of xanthine crystals and uroliths [9,10].

The assessment of microbial protein synthesis and nitrogen balance status in ruminants can be determined through the detection of metabolites in urine. In cattle, the urinary excretion of uric acid and allantoin has been utilized as potential markers for assessing rumen microbial synthesis [11,12,13,14]. In small animals, monitoring purine metabolites can be useful in the early diagnosis of metabolic diseases and in the prevention of urolith formation [15].

In this context, analytical tools for the reliable determination of these endogenous compounds are needed in order to define physiological reference intervals and evaluate the effects of specific therapies (e.g., allopurinol therapy, or dietary and nutritional approaches). In companion animals and large animals, purine measurement is generally performed by colorimetric assays, capillary electrophoresis, conventional liquid chromatography or spectrophotometric methods [5,15,16,17,18,19]. However, a limitation of such approaches is their lack of specificity due to the interference of other endogenous substances.

Liquid chromatography–tandem mass spectrometry (LC–MS/MS) has progressively become a common diagnostic technique in human medicine for therapeutic drug monitoring, endocrinology and toxicology purposes [20,21,22,23,24,25]. Thanks to its unmatched performance, this efficient technique is progressively spreading in veterinary practice as well [26,27,28,29,30]. The use of matrix-matched calibrators is strongly suggested in the analysis of biological fluids by mass spectrometry, especially considering the risk of over- or underestimation deriving from the matrix effect. When quantifying endogenous compounds, the absence of an analyte-free matrix complicates accuracy and precision [31,32,33]. A common approach to preparing calibration curves involves using surrogate matrices, such as neat solvents, water or synthetic/stripped matrices [33]. While neat solvents and water are simple options, they often generate matrix effect-related biases in the quantification of target compounds in unknown samples. To avoid this problem, artificially prepared or analyte-free matrices (which have been stripped of endogenous compounds of interest) can be used, although they might be affected by potential subject-related differences. This can only be prevented through the addition of a stable isotope analog internal standard for each target analyte. However, the main drawbacks of these approaches are the limited commercial availability of surrogate matrices, particularly for non-human species, and their high costs, along with those of the labeled internal standards [32].

Alternatively, a background subtraction approach can be followed: the endogenous concentration of the analytes detected in a calibration curve prepared using individual or pooled biological fluids can be subtracted from the added concentrations in order to quantify unknown samples. The subject-related matrix effect, as well as variable and unpredictable endogenous concentrations, can significantly affect the reliability of this strategy. These critical points can be bypassed with the somehow more laborious standard addition method, where multiple aliquots of each sample are spiked at known levels to build their own calibration curve. The concentration of the investigated analyte is then calculated as the negative x-intercept of the calibration line [31,32,33].

We have recently developed two ultra-high performance liquid chromatography–tandem mass spectrometry (UHPLC-MS/MS) approaches for measuring uric acid and allantoin in canine serum and urine, as well as in bovine urine. The aim of our study was to investigate whether this technique could provide analytical support for research and clinical activities conducted in the veterinary hospital of our university. Our outcomes and the critical points we had to deal with during the method’s development are discussed.

## 2. Materials and Methods

### 2.1. Chemicals and Reagents

Pure analytical standards of uric acid and allantoin were purchased from Toronto Research Chemicals (Toronto, ON, Canada). Acetonitrile, methanol, ammonium formate, ammonium acetate and formic acid (all of LC–MS grade) were purchased from Sigma-Aldrich (St. Louis, MO, USA). Ultrapure water was freshly produced in-house using a Sartorius Arium^®^ Ultrapure Water System (Varedo, MB, Italy).

### 2.2. Standard and Working Solutions

Individual stock solutions of 500 µg/mL (uric acid) and 1000 µg/mL (allantoin) were prepared by dissolving 10 mg of uric acid in 20 mL of water and 10 mg of allantoin in 10 mL of water, both adjusted to pH 12 with sodium hydroxide (Toronto Research Chemicals, Toronto, ON, Canada) in 20 and 10 mL of water adjusted to pH 12 with sodium hydroxide, respectively.

Working solutions containing both analytes at different concentrations were then prepared and used to build eight-point calibration curves. Pure powders of both compounds were stored at −20 °C.

Given the poor stability of the allantoin solutions that emerged during preliminary experiments, all solutions were freshly prepared for each testing day and discarded at the end of the experimental session.

### 2.3. Animal Samples

To investigate the most suitable approach for measuring uric acid and allantoin in both matrices and species, we prepared calibrators and quality control (QC) samples of the two analytes in water with individual samples collected from four different subjects (n = 4 dogs; n = 4 bovines) and in pools of such samples. Serum and urine specimens were obtained from 4 healthy blood donor dogs at the blood bank of the university veterinary hospital (Labrador Retriever, male, 5 years old; mixed breed, female, 7 years old; Leonberger, male, 2 years old; mixed breed, female, 4 years old). Animals were eligible for inclusion if they had no history or signs of recent or chronic illness and had not received any medication, apart from routine vaccinations and prophylactic treatment for ectoparasites or endoparasites. Their health status was confirmed through an unremarkable medical history, a physical examination and a clinicopathological evaluation. During method validation, two additional dogs (Boxer, male, 11 years old; mixed breed, female, 8 years old) suspected by clinicians of having low and high purine metabolite levels were analyzed to ensure the suitability of the analytical approach across a broader concentration range. Bovine urine samples were collected by free catch from 4 healthy Holstein cows (between 2 and 4 years old) at the university stable. These animals were considered healthy based on their history, physical examinations, and unremarkable outcomes in their chemistry profile, complete blood count, and urinalysis. They were fed commercial diets, as documented in their medical records.

The use of these samples was approved by the Ethics Committee for Animal Welfare of the University of Bologna (Protocol No. 306122, dated 2 December 2021, for bovines, and Protocol No. 57790, dated 3 March 2023, for dogs). Informed consent was obtained from the owners of the two dog patients, allowing the use of small aliquots of samples initially collected for diagnostics procedures for research purposes as well. All samples were stored at −80 °C until UHPLC-MS/MS analysis.

### 2.4. Sample Preparation

#### 2.4.1. Serum Samples (Canine)

Ten microliters of serum (or pooled serum or water as the surrogate matrix) were spiked with 40 μL of a mixed working solution or pH 12 aqueous solution to obtain fortification levels of 0, 2.5, 5, 10, 25, 50, 100 and 200 µg/mL for uric acid, and of 0, 10, 20, 50, 100, 200 and 500 µg/mL for allantoin. Protein precipitation was carried out by adding 100 µL of methanol to each sample, agitating in a vortex mixer for 30 sec and then centrifuging at 21,000× *g* for 10 min at 20 °C. Twenty microliters of the supernatant was finally transferred to an LC vial containing 180 μL of 0.1% formic acid aqueous solution, which was then agitated in a vortex mixer.

#### 2.4.2. Urine Samples (Canine and Bovine)

A 20 µL aliquot of 10-fold prediluted urine (or pooled urine or water as a surrogate matrix) was fortified with 80 μL of a mixed working solution or pH 12 aqueous solution to obtain calibrators at 0, 50, 100, 250, 500, 1000, 2500 and 5000 µg/mL for uric acid, and 0, 500, 1000, 2500, 5000, 10,000, 25,000 and 50,000 µg/mL for allantoin (corresponding to 10-fold higher concentrations in undiluted urine). Samples were added to 100 µL of water, agitated in a vortex mixer for 30 s and centrifuged for 10 min at 21,000× *g* at 20 °C. The supernatant was then diluted 40-fold in water. Ten microliters of a sample from each of the vials was injected into the UHPLC-MS/MS system.

### 2.5. LC–MS/MS Analysis

Ultra-high performance liquid chromatography (UHPLC) was performed on a Waters Acquity UPLC^®^ system equipped with a binary pump, thermostatted autosampler, column oven, vacuum degasser and condenser (Waters, Milford, MA, USA). Analyte separation was performed with a Waters HSS T3 (2.1 × 50 mm, 1.8 µm) column maintained at 40 °C and protected by the corresponding VanGuard pre-column (Waters, Milford, MA, USA). The mobile phase consisted of 0.1% formic acid in water (A) and methanol (B) at a variable percentage, flowing at 0.4 mL/min during a 4.5 min run. The program started with 99% A for 0.2 min, switched to 5% A for 0.7 min, then was kept for 1.6 min and finally returned to the initial conditions for 0.7 min before letting the column re-equilibrate for 1.3 min.

The LC system was coupled to a triple quadrupole mass spectrometer (Xevo TQ-S Micro, Waters, Milford, MA, USA), equipped with an electrospray ionization (ESI) source used in positive mode. The capillary voltage was set to −1.5 kV, the desolvation gas flow set to 900 L/hr and the cone gas flow set to 50 L/hr while the source temperature and desolvation temperature were 150 and 500 °C, respectively. Acquisition was performed in multiple reaction monitoring (MRM) mode, selecting the following quantification and confirmation transitions for each analyte: 166.9 > 123.9 *m*/*z* (48 V; 13 eV) and 166.9 > 96.0 *m*/*z* (48 V; 16 eV) for uric acid; 156.9 > 113.9 *m*/*z* (25 V; 11 eV) and 156.9 > 96.9 *m*/*z* (25 V; 14 eV) for allantoin. Data were acquired and processed with the instrument’s proprietary software (MassLynx 4.2, Waters, Milford, MA, USA).

### 2.6. Method Validation

Method validation was conducted following the European Medicines Agency ICH M10 guideline [34] over three separate days of testing on serum, urine and water. The considered parameters, following the acceptance criteria proposed, were specificity, calibration range, accuracy, precision, recovery (RE), matrix effect (ME), carry-over, stability and reinjection reproducibility.

#### 2.6.1. Specificity and Selectivity

The retention times of uric acid and allantoin were determined by injecting pure standards at a concentration of 0.1 µg/mL for each compound. The retention factor (k) for each analyte was calculated as follows:

k = (t_r_ − t_m_)/t_m_

Subsequently, specificity was verified by injecting 10 water samples and mobile phases to confirm that no chromatographic signals were detected at the retention times of the target analytes.

#### 2.6.2. Calibration Range

As described in Section 2.4, calibration curves in pooled serum (individual serum for the standard addition approach) or water, including an unfortified sample containing the endogenous concentration of the two purine metabolites and seven calibrators (2.5, 5, 10, 25, 50, 100 and 200 µg/mL for uric acid, and 0, 10, 20, 50, 100, 200 and 500 µg/mL for allantoin), were prepared during each day of validation.

For urine, calibration curves in pooled urine samples (individual urine for the standard addition approach) or water were prepared at 0, 50, 100, 250, 500, 1000, 2500 and 5000 µg/mL for uric acid, and 0, 500, 1000, 2500, 5000, 10,000, 25,000 and 50,000 µg/mL for allantoin (corresponding to 10-fold higher concentrations in undiluted urine).

The endogenous level of the analyte was calculated as the negative x-intercept of the calibration curve. Background subtraction was applied to ensure the accurate quantification of unknown samples. All calibrators were required to be within ±15% of the expected value, and the correlation coefficient (R^2^) was deemed acceptable if it was ≥0.99.

#### 2.6.3. Lower Limit of Quantification (LLOQ)

The method’s lower limit of quantification (LLOQ) was determined as the lowest endogenous concentration measured producing a signal-to-noise (S/N) ratio ≥10 with adequate accuracy (±20%) and precision (<20%) following the analysis of four replicates. A calibration curve in water, used as a surrogate matrix, was also processed during each session to verify if the quantification of the two compounds in the unknown samples was comparable; the results were obtained with matrix-matched calibration curves.

#### 2.6.4. Accuracy and Precision

To evaluate intra- and inter-day accuracy and precision for both methods, QC samples were prepared at three different spiked levels: 2.5, 10 and 50 µg/mL for uric acid and 10, 50 and 200 µg/mL for allantoin in serum; at 50, 250 and 2500 µg/mL for uric acid and 500, 2500 and 10,000 µg/mL for allantoin in urine. These QCs were prepared in triplicates alongside the calibration curve in each matrix for both species during the 3 days of validation.

Accuracy, defined as the relative difference between found and expected levels, had to be lower than ±15%. Precision, expressed by the coefficient of variation (CV%) among multiple individual measures, was considered acceptable if it was <15% for each QC level. For both matrices, those parameters were also evaluated in water and in the pooled matrices, preparing QCs at the same three spiked levels.

#### 2.6.5. Matrix Effect (ME)

The matrix effect was evaluated by applying the standard addition approach to the individual calibration curves prepared in serum and urine collected from each of the four dogs or bovines. Initially, the endogenous concentrations of the analytes were determined. Subsequently, the unfortified sample of each subject was quantified on the calibration curves of the other three subjects and on the pooled matrix-matched calibration curve. Furthermore, the mean slopes of the curves in the water and pooled matrices were compared to those in the two biological fluids from individual patients.

#### 2.6.6. Recovery

The recovery of the method was determined by spiking two series of aliquots (n = 6 for both series) at three different concentrations, one before and one after the extraction, and then the mean measured values were compared. The concentrations tested for the recovery were as follows: 5, 10 and 25 µg/mL for uric acid and 10, 20 and 50 µg/mL for allantoin.

Recovery was determined as the percentage ratio between the analyte response in a biological sample spiked before extraction and that in a sample spiked after extraction.

#### 2.6.7. Stability

The stability of uric acid and allantoin was assessed in fresh pooled serum samples from healthy blood donors (n = 4, analyzed in triplicate), as well as fresh urine samples collected from healthy (n = 4, analyzed in triplicate) and diseased (n = 2, analyzed in triplicate) subjects. Short-term stability was evaluated under two conditions: room temperature (20 °C) and refrigerator temperature (4 °C) over 24, 48 and 72 h. To assess long-term stability, samples were stored at −20 °C and −80 °C for 1, 3 and 5 months. Additionally, the stability of extracted serum and urine samples was evaluated after being left at 20 °C in the autosampler for 24 h and after freezing at −20 °C for 24 h.

This assessment involved comparing the mean measured concentration (baseline concentration) on day 0 with concentrations found after storage. The results were expressed as the percentage variation between the concentrations measured after the different storage times and the baseline concentration on day 0. Each compound was considered stable in serum and urine samples when the mean measured concentrations fell within ±15% of the nominal concentration.

#### 2.6.8. Carry-Over

To assess the absence of carry-over, mobile phases were analyzed following the injection of the highest calibrators of both matrices. Carry-over following the high concentration calibrator should not be greater than 20% of the LLOQ.

## 3. Results

The developed UHPLC-MS/MS approaches were validated in accordance with the current European Medicines Agency guidelines [34]. The retention time and retention factor (k) were 0.41 min and 0.21, respectively, for allantoin, and 0.62 min and 0.82, respectively, for uric acid.

**Figure 2:**
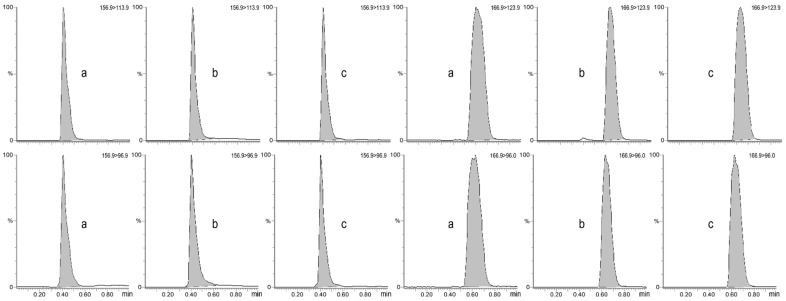
Chromatograms of the product ions used for quantification (**top**) and confirmation (**bottom**) for allantoin (**left**) and uric acid (**right**), obtained from the analysis of dog serum (a), dog urine (b) and bovine urine (c) collected from healthy subjects.

The selectivity was demonstrated by mass chromatograms, which showed no interference from endogenous compounds within the same time window as uric acid and allantoin in both matrices (Figure 2). The specificity of the method was proven by the absence of interfering peaks at the retention times of the two compounds under study after the injection of pure water.

The LLOQ in dog serum was 2.5 µg/mL for uric acid and 10 µg/mL for allantoin. In canine and bovine urine, the LLOQ was 50 µg/mL for uric acid and 500 µg/mL for allantoin. In both species, all calibration curves for uric acid and allantoin in serum and urine were linear, with a coefficient of determination of R^2^ > 0.99, and all calibration levels were within ±15%. The accuracy of the two methods (always within ±15%) and precision (always <15%) were evaluated by preparing QC samples in quadruplicate at three different concentrations. The data are shown in Table 1 and Table 2.

The presence of ME when using calibration curves from different subjects, as well as applying water and background subtraction methods, led to quantification discrepancies and subsequent misleading results. The complete evaluation of ME obtained for dogs is reported in Table 3.

For both analytes, the comparison between samples spiked before and after extraction showed recoveries in the serum between 98 and 104%.

The repeated injection of mobile phases after the ULOQ did not generate any detectable chromatographic signal, confirming the absence of carry-over. Short-term stability tests showed that uric acid in serum remained stable at 4 °C and 20 °C for 24 h but became unstable after 48 and 72 h, with deviations of up to ±22% and ±40%, respectively. In contrast, long-term assessments indicated that uric acid remained stable for 1, 3 and 5 months at −20 °C and −80 °C, with variations below ±15%. Allantoin in serum was stable for 48 h at both refrigerator and room temperatures but became unstable after 72 h under both conditions, with deviations of up to ±26%. Additionally, long-term storage at −20 °C and −80 °C resulted in allantoin instability, with deviations already around ±50% after 1 month.

In urine samples, both uric acid and allantoin were unstable in all short-term stability tests conducted, with deviations of up to ±39%. However, they remained stable when stored at both −20 °C and −80 °C for up to 5 months, with deviations below ±13.5%.

For both analytes and matrices, differences within ±5% were observed when reanalyzing extracted samples kept in the autosampler at 5 °C for 24 h, and similar differences within ±5% were found for extracted samples stored at −20 °C.

## 4. Discussion

During method development, chromatographic columns packed with different stationary phases (Acquity UPLC BEH C18, BEH HILIC, HSS T3, and Amide, Waters, Milford, MA, USA) and mobile phase compositions were tested on a binary pump (Acquity UPLC, Waters, Milford, MA, USA). Additives such as ammonium formate and ammonium acetate in water, along with different percentages of formic acid, were tested in combination with organic solvents like acetonitrile or methanol. The best peak shape and chromatographic separation for uric acid and allantoin was obtained using 0.1% formic acid in water and methanol under programmed conditions, flowing at 0.4 mL/min during a 4.5 min run.

After optimizing the MS parameters for each target compound in both positive (ESI+) and negative ionization modes (ESI-), we chose the latter due to the higher signal-to-noise ratio obtained.

The presence of ME was first indicated by the difference in mean slopes for calibration curves in water compared to those in the two biological fluids, highlighting the need for matrix-matched calibrators for reliable quantification. To determine the endogenous concentrations of the analytes, we applied the standard addition approach to the individual calibration curves prepared in the serum and urine collected from each of the four dogs. Additionally, unfortified samples of each subject (dog or bovine) were quantified using the calibration curves of the other three subjects, and on the pooled matrix-matched calibration curve. Notably, relevant discrepancies were observed across all subjects, suggesting that using methods other than standard addition to measure unknown samples could lead to misleading results (Table 2 reports the example involving dogs). Therefore, we concluded that standard addition is the most reliable approach for the quantification of uric acid and allantoin in dog serum and urine, as well as in bovine urine, since the recovery and matrix effect are the same between samples and calibrators. This method clearly implies that a large number of samples must be processed, which might require a considerable amount of matrix and is highly time-consuming.

The strength of the proposed protocol lies in its simplicity and efficiency: less than 100 µL of each matrix proved sufficient for the analysis, making it highly practical for laboratory use. The procedure is straightforward with a short run time, allowing for its easy implementation in routine workflow. Another significant advantage is the ability to measure multiple compounds simultaneously, with the individual concentration range being a critical factor for accurate results. The endogenous levels of each analyte play a key role in this, while the increase in the peak area after spiking should be at least 15–20% [32,33].

In our experiment, for both canine serum and urine, as well as for bovine urine, a single injection allowed us to monitor the two analytes. Furthermore, in order to increase the cost- and time-effectiveness, we tested five-point calibration curves (with an un-spiked matrix and four concentrations) along with two QC levels in both serum and urine, verifying that the same results can also be obtained with a less laborious procedure.

Multiple methods [23,35,36,37] using standard addition to measure endogenous analytes in biological fluids are described in the literature. The same approach was also applied during the determination of exogenous compounds to overcome the presence of different matrix effects in different samples [38,39,40]. Thanks to the unmatched selectivity of mass spectrometry, the developed standard addition-based protocol enables enhanced purine metabolite quantification in veterinary medicine and supports clinical diagnostics in defining reliable reference intervals. The possibility to avoid the use of labeled internal standards [36], and the simple and quick sample treatment, also make it more cost-effective.

The overall performance of this standard addition protocol for the determination of uric acid and allantoin in dog serum and urine, as well as in bovine urine, make it not only suitable for research purposes but also for routine use in the laboratory (e.g., in the monitoring of diseases or diet-related purine alterations), even for the analysis of a single subject.

This preliminary application demonstrates the successful measurement of purine metabolites by liquid chromatography–tandem mass spectrometry in these two species. Both uric acid and allantoin were detectable in all analyzed subjects, and the validated calibration range proved suitable for measuring actual concentrations in both healthy and pathological individuals. Furthermore, a single equine plasma sample was also successfully processed, suggesting the suitability of the method for additional animal species. The findings also underscore the potential of this technique as a valuable tool for accurate quantification in veterinary laboratories and clinical settings. Its increasing diffusion, along with continuous technological progress, is leading to more economically sustainable instruments, paving the way for many other similar applications.

However, our study has some limitations. First, while we have identified a reliable strategy for allantoin and uric acid quantification in veterinary medicine using LC–MS/MS, the number of subjects involved in the study was small. In future studies, we plan to apply the method to a more substantial number of samples (serum and urine) collected from healthy dogs (40 ≤ x ≤ 120) to establish reference intervals [41] for uric acid and allantoin using the standard addition method. Subsequently, we intend to extend the application of the method to samples collected from patients affected by diseases known to potentially increase purine metabolism. Another limitation of this study is the absence of a comparison between our results, obtained using the standard addition approach and the LC–MS/MS, and with other conventional methods for uric acid measurement in serum and urine. In the future, we plan to conduct this comparison for all the subjects involved. Another aspect that we can consider adding in the future is the extension of the method to include other purine metabolites and drugs, such as allopurinol, to further enhance its applicability for research studies and clinical routine monitoring and evaluations. Similarly, for bovines, our aim is to apply the method to a larger number of urine samples collected from healthy subjects to also establish reference intervals in this species and in groups fed different diets with different protein contents. Furthermore, recognizing the significance of storage conditions for stability [13,14,18], we plan to conduct stability tests for long-term conditions at −20 and −80 °C and in neutral or acidic environments, enabling us to effectively organize the analysis time in research projects related to this species.

Finally, we plan to extend the validation to horses, where our preliminary results were satisfactory, and other ruminants, such as goats and sheep.

## 5. Conclusions

This is, to our knowledge, the first method for the determination of uric acid and allantoin in dog serum and urine, as well as in bovine urine, using UHPLC-MS/MS with a standard addition approach. Its overall performance makes it not only suitable for research purposes but also for routine laboratory use (e.g., in the monitoring of diseases or diet-related purine alterations), even for the analysis of a single subject. This preliminary study demonstrates the successful measurement of purine metabolites by liquid chromatography–tandem mass spectrometry in various species. The findings also underscore the potential of this technique as a valuable tool for accurate quantification in veterinary clinical diagnostics. Its increasing diffusion, along with continuous technological progress, is leading to more economically sustainable instruments, enabling many other similar applications.

## Figures and Tables

**Table 1:** Intra-day and inter-day accuracy and precision of uric acid and allantoin in matrix-matched (pooled canine serum and urine) QC samples.

	Dog Serum				Dog Urine			
	Uric Acid		Allantoin		Uric Acid		Allantoin	
	QC1 (2.5 µg/mL)		QC1 (10 µg/mL)		QC1 (50 µg/mL)		QC1 (500 µg/mL)	
Day	Accuracy (%)	Precision (CV%)	Accuracy (%)	Precision (CV%)	Accuracy (%)	Precision (CV%)	Accuracy (%)	Precision (CV%)
1 (n = 3)	−6.3	7.9	1.3	6.4	−0.5	0.8	3.8	6.3
2 (n = 3)	12.2	9.6	−3.2	1.0	−3.6	6.4	13.8	10.3
3 (n = 3)	−7.7	13.3	1.6	6.7	−3.7	6.3	4.7	5.6
Inter-day (n = 9)	−0.6	13.2	−0.1	5.1	−2.6	4.7	7.4	8.2
	QC2 (10 µg/mL)		QC2 (50 µg/mL)		QC2 (250 µg/mL)		QC2 (2500 µg/mL)	
Day	Accuracy (%)	Precision (CV%)	Accuracy (%)	Precision (CV%)	Accuracy (%)	Precision (CV%)	Accuracy (%)	Precision (CV%)
1 (n = 3)	−2.1	5.1	6.7	4.7	0.1	0.4	2.6	4.4
2 (n = 3)	−3.9	9.2	0.3	2.8	−3.6	3.2	5.4	7.3
3 (n = 3)	−3.6	12.4	−2.2	2.9	−2.6	3.4	6.6	8.1
Inter-day (n = 9)	−3.2	7.9	1.6	5.1	−2.1	2.9	4.9	6.1
	QC3 (50 µg/mL)		QC3 (200 µg/mL)		QC3 (2500 µg/mL)		QC3 (10,000 µg/mL)	
Day	Accuracy (%)	Precision (CV%)	Accuracy (%)	Precision (CV%)	Accuracy (%)	Precision (CV%)	Accuracy (%)	Precision (CV%)
1(n = 3)	4.7	2.1	4.4	4.6	2.9	7.6	5.4	5.8
2 (n = 3)	2.3	3.4	−0.8	1.1	−10.9	14.1	5.0	6.1
3 (n = 3)	0.7	4.8	−2.1	2.2	−8.0	14.6	5.5	9.7
Inter-day (n = 9)	2.6	3.5	0.5	4.3	−5.3	12.5	5.3	6.4

**Table 2:** Intra-day and inter-day accuracy and precision of uric acid and allantoin in matrix-matched (pooled bovine urine) QC samples.

	Bovine Urine			
	Uric Acid		Allantoin	
	QC1 (50 µg/mL)		QC1 (500 µg/mL)	
Day	Accuracy (%)	Precision (CV%)	Accuracy (%)	Precision (CV%)
1 (n = 3)	−5.9	5.5	−8.3	7.1
2 (n = 3)	0.6	6.6	−8.4	5.8
3 (n = 3)	0.0	13.2	−5.6	1.5
Inter-day (n = 9)	−1.8	8.6	−7.4	4.8
	QC2 (250 µg/mL)		QC2 (2500 µg/mL)	
Day	Accuracy (%)	Precision (CV%)	Accuracy (%)	Precision (CV%)
1 (n = 3)	−3.6	3.3	−0.8	2.0
2 (n = 3)	−3.7	10.1	−4.9	3.2
3 (n = 3)	1.5	7.1	−1.4	1.9
Inter-day (n = 9)	−1.9	6.9	−2.4	2.8
	QC3 (2500 µg/mL)		QC3 (10,000 µg/mL)	
Day	Accuracy (%)	Precision (CV%)	Accuracy (%)	Precision (CV%)
1 (n = 3)	−5.1	7.2	0.1	1.9
2 (n = 3)	−0.4	3.7	0.5	4.5
3 (n = 3)	−1.6	1.4	0.4	4.6
Inter-day (n = 9)	−2.4	4.6	0.3	3.4

**Table 3:** Comparative analytical results for uric acid and allantoin in both the serum and urine of four dogs, obtained using three different quantification approaches. The absolute value is reported for the quantification using the standard addition approach. For the quantification using water as a surrogate matrix and for the background subtraction, the percentage of variation compared to the standard addition is indicated in italics.

*Serum*	Standard Addition	Water	Background Subtraction		
Uric Acid (µg/mL)					
Dog 1	3.3	1.6 *(−51%)*	2.3 *(−29%)*	2.3 *(−29%)*	2.5 *(−23%)*
Dog 2	3.9	2.7 *(−31%)*	3.7 *(−6%)*	4.0 *(+2%)*	6.9 *(+76%)*
Dog 3	4.9	3.4 *(−30%)*	4.9 *(+1%)*	5.1 *(+5%)*	8.7 *(+79%)*
Dog 4	4.6	2.9 *(−36%)*	4.1 *(−10%)*	4.1 *(−10%)*	7.5 *(+65%)*
Allantoin (µg/mL)					
Dog 1	14.9	12.5 *(−16%)*	19.4 *(+30%)*	19.9 *(+33%)*	22.6 *(+51%)*
Dog 2	22.8	12.2 *(−47%)*	19.3 *(−15%)*	21.9 *(−4%)*	17.2 *(−25%)*
Dog 3	20.8	13.1 *(−37%)*	19.9 *(−5%)*	23.2 *(+11%)*	18.4 *(−12%)*
Dog 4	18.9	10.4 *(−45%)*	16.5 *(−13%)*	16.3 *(−14%)*	14.7 *(−22%)*
***Urine***	**Standard Addition**	**Water**	**Background Subtraction**		
Uric Acid (µg/mL)					
Dog 1	530.2	672.9 (+27%)	650.2 (+23%)	737.1 (+39%)	653.1 (+23%)
Dog 2	535.4	473.6 (−11%)	562.6 (+5%)	460.3 (−14%)	460.0 (−14%)
Dog 3	199.2	22.0 (−89%)	287.0 (+44%)	244.2 (+23%)	264.2 (+33%)
Dog 4	76.0	100.3 (+32%)	118.4 (+56%)	97.0 (+28%)	109.2 (+44%)
Allantoin (µg/mL)					
Dog 1	7276.8	7916.0 (+9%)	8543.1 (+17%)	7528.2 (+3%)	5979.0 (−18%)
Dog 2	8967.1	7090.4 (−21%)	6992.3 (−22%)	5358.2 (−40%)	8543.0 (−5%)
Dog 3	5087.3	5101.3 (0%)	5027.4 (−1%)	3854.4 (−24%)	5027.4 (−1%)
Dog 4	1871.7	2673.0 (+43%)	2633.7 (+41%)	2873.0 (+53%)	2542.0 (+36%)

## Data Availability

All data is contained within the article.
